# Effect of COVID-19 inactivated vaccine on anti-Müllerian hormone in Chinese women: a retrospective cohort study

**DOI:** 10.3389/fendo.2025.1403722

**Published:** 2025-06-04

**Authors:** Mingjie Bao, Leizhen Xia, Yan Ling, Quan Wen, Xin Shen, Ting Wang, Si Qian, Liqun Wang, Changhua Wang, Shiwei Peng, Yongping Zhang, Shaoping Zhong, Hongying Xu, Yuan Zhu

**Affiliations:** ^1^ Department of Obstetrics and Gynecology, Jiangxi Provincial People's Hospital, The First Affiliated Hospital of Nanchang Medical College, Nanchang, China; ^2^ Department of Reproductive Medicine, Jiangxi Maternal and Child Health Hospital, Nanchang, China; ^3^ Department of Gynecology, Jiangxi Maternal and Child Health Hospital, Nanchang, China; ^4^ Department of Clinical Medicine, Nanchang University, Nanchang, China

**Keywords:** COVID-19 vaccine, COVID-19 inactivated vaccine, anti-Müllerian hormone, ovarian reserve, reproductive health

## Abstract

**Introduction:**

This study aimed to assess the impact of inactivated COVID-19 vaccine on Anti-Müllerian hormone (AMH) levels in Chinese women.

**Methods:**

A retrospective analysis was conducted on women aged 18-45 who had undergone two AMH tests between March 2020 and September 2021. Participants were grouped based on vaccine doses (two- and three-dose), time intervals since vaccination, and manufacturers. The difference in AMH levels and the percentage changes in AMH were measured.

**Results:**

The results revealed no significant differences in AMH levels between the vaccinated groups (two- and three-dose) and the control group, both in unadjusted and adjusted analyses. Subgroup analysis showed no statistical difference in either absolute or percentage changes of AMH levels among different time-interval groups and manufacturer groups.

**Discussion:**

In conclusion, the number of doses, time interval, and manufacturer of the inactivated COVID-19 vaccine did not affect AMH levels in Chinese women.

## 1 Introduction

Coronavirus disease 2019 (COVID-19), or the “new coronavirus pneumonia,” is a respiratory infectious disease that has been spreading rapidly worldwide since December 2019. The COVID-19 epidemic is the most severe global public health outbreak since World War II, and it seriously threatens human health. As the first country to be hit by the COVID-19 epidemic (1), China is the best place for the research on the novel coronavirus and its vaccines. The Chinese government announced the lifting of epidemic control on December 7, 2022, implying that the focus of epidemic prevention and control has shifted from controlling the source of infection and blocking transmission routes to the direction of protecting susceptible populations, representing a need for more people to participate in vaccination in the face of a raging epidemic, resulting in a surge in demand for vaccines. Driven by policy support and media coverage, the vast majority of Chinese residents choose to be vaccinated against the COVID-19 epidemic. However, young people who are planning to become pregnant are hesitant to receive vaccination because of the concern about the safety of the vaccination. A survey in 2023 showed that the COVID-19 vaccination rate of men and women preparing for pregnancy was significantly lower than the average vaccination rate in China (2). On the other hand, some people who have been vaccinated also worry about the harm to their physical health. Among these concerns, apprehensions regarding reproductive health are notably prevalent.

First, some evidence supports that coronaviruses may have an impact on human reproductive health. COVID-19 is caused by SARS-CoV-2 pathogen infection (3–6), a single positive-stranded RNA coronavirus with regularly arranged spines on the envelope. The virus binds to angiotensin-converting enzyme 2 (ACE2), mediated by the viral surface spine glycoprotein (S protein), to enter cells (6, 7). ACE2 has been detected in human tissues of different organs, including the heart, kidney, intestine, and blood vessels. ACE2 has also been detected in organs related to reproduction, such as ovaries, uterus, vagina, placenta, and testes (8, 9). Based on the considerable regulatory role of ACE2 on reproduction (10, 11), SARS-CoV-2 may affect female reproductive function by affecting ACE2. Studies have shown that SARS-CoV-2 affects ovarian reserve in women. A study by Ding et al. in March 2021 showed that women infected with COVID-19 had lower Anti-Müllerian hormone (AMH) levels, higher FSH levels, and higher levels of testosterone and prolactin than healthy women (12).

Secondly, a vaccine is a biological agent derived from a virus. If a virus exerts a specific effect on the body, it is plausible that the vaccine may elicit similar effects. Since the outbreak of COVID-19, many types of vaccines, such as mRNA vaccines, DNA vaccines, inactivated vaccines, recombinant protein subunit vaccines, virus vector vaccines, and virus-like particle vaccines, have been used. Studies have shown that other new crown vaccines, such as mRNA vaccines, impact women’s ovarian reserve (13–15). Inactivated vaccines, widely administered in China, are known to retain the intact structure of the virus, so inactivated vaccines may be more likely to cause damage to reproductive health than other types of vaccines. However, current research on the impact of inactivated vaccines on female reproductive health in China is rather limited.

Therefore, the purpose of this study was to investigate whether the COVID-19 vaccination of inactivated vaccines in China would affect AMH in Chinese women, and thus indirectly assess whether it would affect ovarian function in Chinese women.

## 2 Methods

### 2.1 Subjects

This study was a retrospective study of patients admitted to a provincial tertiary hospital in China from March 2020 to September 2021. Informed consent was obtained from all subjects. Inclusion criteria are as follows: female, aged between 18 and 45; received two or more AMH tests between March 2020 and September 2022; the first AMH was within the normal range (16). The exclusion criteria were as follows: postmenopausal women, those with polycystic ovarian syndrome, those who were pregnant, and those who had ovarian surgery during this period. The cases with incomplete information were excluded in our analysis.The cases with incomplete information were excluded in our analysis. This study was approved by the Ethics Committee of Jiangxi Provincial Maternal and Child Health Hospital (approval number: EC-KT-202309). We certify that the study was performed in accordance with the 1964 declaration of HELSlNKl and later amendments.

### 2.2 Vaccination strategy

The vaccination strategy in China is as follows: voluntary principle, available to people ≥ 18 years, with two doses routinely administered by intramuscular injection into the deltoid muscle of the upper arm, and the interval between the two doses should be ≥ 3 weeks but ≤ 8 weeks. The third dose (booster) should not be given until 6 months after the second dose. If the vaccination is not completed in accordance with the procedure, making up the vaccination as soon as possible is recommended. Patients who received Sinopharm vaccine or Sinovac vaccine were included in this study, and some patients who received both vaccines were also included in this study. Vaccination information from official immunization records was collected in a personal mobile application (app).

### 2.3 Research grouping criteria

In this study, the subjects were divided into three groups in accordance with the number of doses received and whether they received the vaccine: a two-dose group (two doses received), a three-dose group (three doses received), and control group (no vaccination due to voluntary principle). From March 2020 to September 2022, women who received two or 3 doses of the vaccine and were tested for AMH before the first dose and after the last dose were included in the two- or three-dose vaccine group. During the same period, women who underwent two AMH tests at the research hospital and had never been vaccinated were included in the control group. In current studies focusing on the effects of inactivated vaccines on AMH, participants who received two doses of the vaccine were included as subjects (17). In addition to investigating the effects of two doses of vaccination, this study also incorporated individuals who received booster shots (three-dose groups). Due to the novelty of designing two vaccine regimens and the uncertainty surrounding the effect of vaccination on AMH levels, the sample size could not be predetermined.

**Figure 1:**
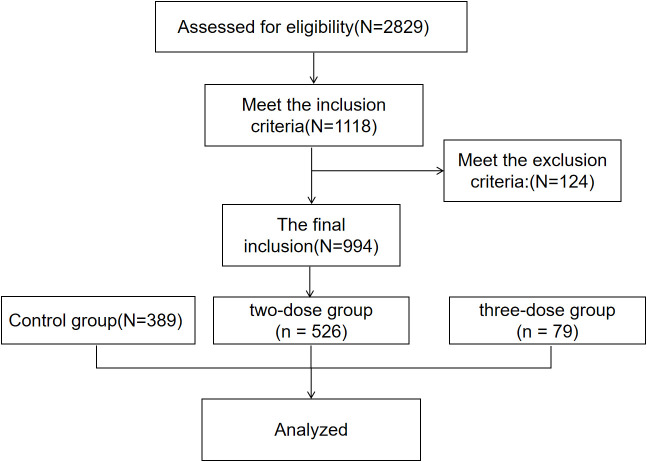
Flow chart showing the design, inclusion and exclusion criteria of patients in the study. The Inclusion criteria: female, aged between 18 and 45; received two or more AMH tests between March 2020 and September 2022; the first AMH was within the normal range. The exclusion criteria: postmenopausal women, those with polycystic ovarian syndrome, those who were pregnant, and those who had ovarian surgery during this period.

In the end, 526, 79, and 389 women were included in the two-dose, three-dose, and control groups, respectively (**Figure 1**).

### 2.4 Study indicators

AMH was measured by Elecsys^®^AMH Plus immunoassay in this provincial tertiary hospital in China. After taking venous blood at the blood sampling window, the serum was obtained by centrifugation by experienced laboratory staff, and the serum was obtained by Cobas e 801 analyzer 127 (Roche Diagnostics, Switzerland) for testing.The study metrics were as follows: the difference in AMH (last AMH – first AMH) and the percentage change in AMH [(last AMH – first AMH)/first AMH)].

### 2.5 Statistical methods

SAS 9.4 software was applied for statistical analysis. Count data were described by frequencies or percentages, and the chi-square test was applied for comparisons. The measurement data were tested for normality by the Shapiro–Wilk test. The data conforming to a normal distribution were compared by t-test and expressed as mean ± standard deviation (x ± s), whereas those not conforming to a normal distribution were expressed as median P50 (25th percentile, P25; 75th percentile, P75) and compared by Kruskal–Wallis rank sum test. The AMH change values were used in the Wilcoxon signed-rank test. A generalized linear model was applied for multivariate analysis of AMH change values. p < 0.05 was considered a statistically significant difference.

## 3 Results

### 3.1 The baseline characteristics of the study participants

**Table 1** shows the baseline characteristics of the study participants. The data were presented in the form of median after normality test. Among the 994 women included in the study, significant differences were observed in terms of median age, first AMH level, and the time interval between AMH (days) in the two-dose (n = 526) and three-dose (n = 79) groups compared with the control group (n = 389). Due to the differences in the underlying information, multifactorial analysis was applied to adjust the data for the following statistical analysis to increase the credibility of the study results.

**Table 1:** Demographic characteristics of the study population.

Variables	Two-dose (n = 526)	Three-dose (n = 79)	Control group (n = 389)	P-value 1^*^	P-value 2^#^
Age (years)	32.67 (28.78,37.3)	33.76 (29.04,39.44)	31.33 (28.1,35.18)	0.002	0.002
Classification				0.023	0.009
<30 years (%)	169 (32.1)	21 (26.6)	149 (38.3)		
30–34 years (%)	176 (33.5)	24 (30.4)	138 (35.5)		
≥35 years (%)	181 (34.4)	34 (43)	102 (26.2)		
First AMH (ng/mL)	2.44 (1.12,4.21)	1.89 (0.82,3.71)	2.63 (1.35,4.8)	0.043	0.001
Last AMH (ng/mL)	2.16 (0.97,4.09)	1.32 (0.66,3.03)	2.52 (1.14,4.58)	0.020	0.000
Time interval between AMH examinations (days)	335 (238,432)	429 (355,556)	221 (132,329)	<.001	<.001
Classification				<.001	<.001
Within 6 months	70 (13.3)	0 (0)	147 (37.8)		
7 to 12 months	239 (45.4)	22 (27.9)	175 (45)		
13 to 18 months	183 (34.8)	36 (45.6)	58 (14.9)		
More than 18 months	34 (6.5)	21 (26.6)	9 (2.3)		

*Two-dose vs. Control group; # Three-dose vs. Control group. Shapiro-Wilk test and Kruskal-Wallis rank sum test were used for statistical analysis.

### 3.2 Difference and percentage change in AMH among the three groups

As illustrated in **Table 2**, compared with the control group, the two-dose (−0.14 vs. −0.07, P = 0.332; −8% vs. −5%, P = 0.322) and three-dose groups (−0.17 vs. −0.07, P = 0.303; −14% vs. -5%, P = 0.073) showed non-statistically significant difference in the difference and percentage change in AMH, respectively.

**Table 2:** Comparison of the difference in AMH and the percentage change in AMH among the 3 groups.

Outcome Measures	Two-dose group (n = 526)	Three-dose group (n = 79)	Control group (n = 389)	P-value 1^*^	P-value 2^#^
The difference in AMH (ng/mL)	−0.14 (−0.64, 0.33)	−0.17 (−0.58, 0.17)	−0.07 (−0.66, 0.51)	0.332	0.303
The percentage change in AMH (%)	−8 (−30, 18)	−14 (−37, 12)	−5 (−28, 25)	0.322	0.073

^*^two-dose group versus control group; ^#^three-dose group versus control group. The difference in AMH:last AMH – first AMH; The percentage change in AMH:(last AMH – first AMH)/first AMH. Shapiro-Wilk test and Wilcoxon signed-rank test were used for statistical analysis.

**Table 3** also shows no significant difference in the difference in AMH between the two/three-dose groups and the control group after adjusting for the first AMH, age, and time interval between AMH examinations by using a generalized linear model for the analysis (P = 0.630; P = 0.416). In addition, the percentage change in AMH (P = 0.515; P = 0.651) was not statistically significant.

**Table 3:** Generalized linear models of AMH change.

Parameter	B (95% CI)	SE	Wald Chi-square	P-value
The difference in AMH (ng/mL) between two-dose group and control group				
Two-dose group versus control group	−0.04 (−0.2, 0.12)	0.0826	0.231	0.630
First AMH	−0.13 (−0.16, −0.09)	0.0179	48.632	< 0.001
Age (years)	−0.04 (−0.06, −0.03)	0.0077	27.024	< 0.001
Time interval between AMH examinations (days)	0 (0, 0)	0.0003	0.391	0.532
The percentage change in AMH (%) between two-dose group and control group				
Two-dose group versus control group	−9 (−34,17)	0.1311	0.424	0.515
First AMH	−0.09 (−0.14, −0.03)	0.0283	9.25	0.002
Age (years)	−0.01 (−0.03, 0.02)	0.0121	0.346	0.556
Time interval between AMH examinations (days)	0 (0, 0)	0.0005	3.126	0.077
The difference in AMH (ng/mL) between three-dose group and control group				
Three-dose group versus control group	−0.14 (−0.49, 0.2)	0.1754	0.66	0.416
First AMH	−0.13 (−0.19, −0.08)	0.0263	25.878	0.000
Age (years)	−0.05 (−0.07, −0.03)	0.0113	19.902	0.000
Time interval between AMH examinations (days)	0 (0, 0)	0.0004	0	0.991
The percentage change in AMH (%) between three-dose group and control group				
Three-dose group versus control group	−16 (−83, 52)	0.3434	0.205	0.651
First AMH	−0.12 (−0.22, −0.02)	0.0515	5.094	0.024
Age (years)	−0.01 (−0.05, 0.04)	0.0222	0.161	0.688
Time interval between AMH examinations (days)	0 (0, 0)	0.0008	1.167	0.280

Generalized linear model was used for statistical analysis.

### 3.3 Effect of time interval on the difference and percentage change in AMH

**Table 4a** and **Table 4b** shows the influence of the time interval from the last vaccine injection to the last AMH examination over the difference in AMH and the percentage change in AMH. All patients in the two-dose group were grouped by the time interval from the last vaccine injection to the last AMH examination received, and they were divided into four groups of up to 0–60 days (n = 135), 61-120days (n = 154), 121–180 days (n = 146), and more than 180 days (n = 91). The median age of patients and the first-time AMH median values were not significantly different among the four groups. The results of the statistical analysis found no significant change in the difference and percentage change in AMH among the four groups. By using data within 0–60 days as a reference, the difference in AMH adjusted β values (95% CI) of the other groups were −0.01 (−0.26, 0.24), −0.16 (−0.42, 0.09), and −0.25 (−0.55, 0.06), respectively, and the adjusted β values (95% CI) for the percentage change in AMH were 0.1 (−0.13, 0.32), −0.03 (−0.26, 0.2), and −0.02 (−0.29, 0.25), there was no statistical difference.

**Table 4a:** Statistical characteristics of different AMH time intervals in the 2-dose group.

Variables	0–60 days (n = 135)	61–120 days (n = 154)	121–180 days (n = 146)	≥181 days (n = 91)	P-value
Age (years)	32.68 (28.32, 36.52)	32.74 (29.3, 38.69)	32.9 (29.3, 37.43)	31.53 (27.72, 35.88)	0.143
First AMH (ng/mL)	2.58 (1.32, 4.28)	2.13 (0.93, 3.86)	2.62 (0.99, 4.55)	2.49 (1.09, 4.18)	0.465
Last AMH (ng/mL)	2.26 (1.28, 4.12)	1.86 (0.91, 3.89)	2.27 (0.83, 4.23)	1.94 (0.86, 4.03)	0.662
Time interval between AMH examinations (days)	276 (166, 369)	324 (212, 409)	348.5 (257, 451)	406 (314, 507)	< 0.001

^*^adjusted factors: Age, First AMH, Time interval between AMH examinations. Shapiro-Wilk test and Kruskal-Wallis rank sum test were used for statistical analysis.

**Table 4b:** Effect of the time interval on the difference in AMH and the percentage change in AMH in the two-dose group.

Parameters	0–60 days (n = 135)	61–120 days (n = 154)	121–180 days (n = 146)	≥181 days (n = 91)	P-value
The difference in AMH (ng/mL)	−0.03 (−0.63, 0.44)	−0.12 (−0.61, 0.34)	−0.21 (−0.63, 0.23)	−0.1 (−0.75, 0.39)	0.403
Crude B (95% CI)	–	0.01 (−0.25, 0.26)	−0.17 (−0.43, 0.09)	−0.22 (−0.51, 0.07)	
Adjust B (95% CI)^*^	–	−0.01 (−0.26, 0.24)	−0.16 (−0.42, 0.09)	−0.25 (−0.55, 0.06)	
The percentage change in AMH (%)	0 (−28, 30)	−7 (−30, 20)	−9 (−30, 12)	−10 (−32, 15)	0.202
Crude B (95% CI)	–	0.08 (−0.14, 0.31)	−0.09 (−0.31, 0.14)	−0.1 (−0.36, 0.16)	
Adjust B (95% CI)^*^	–	0.1 (−0.13, 0.32)	−0.03 (−0.26, 0.2)	−0.02 (−0.29, 0.25)	

The difference in AMH:last AMH – first AMH; The percentage change in AMH:(last AMH – first AMH)/first AMH. Rank sum test and regression analysis were used for statistical analysis.

### 3.4 Effect of vaccine manufacturers on the difference in AMH and the percentage change in AMH

**Table 5a** and **Table 5b** shows the effect of vaccine manufacturers on AMH. In this study, the vaccine manufacturers in the two-dose group were analyzed, which included China National Pharmaceutical Group Co. Ltd. (Sinopharm vaccine) and Sinovac Life Sciences Co., Ltd. (Sinovac vaccine). The participants were further divided in accordance with the vaccine manufacturer: Sinopharm group (Sinopharm Vaccine only, n = 129), Sinovac group (Sinovac vaccine only, n = 153), and a mixed group (inoculated against Sinopharm and Sinovac vaccines, n = 244). The results found no significant difference in the AMH difference and the percentage change in AMH among these three groups. As shown in **Table 5b**, with Sinopharm as the reference, the adjusted β values (95% CI) for the difference in AMH were −0.05 (−0.3, 0.2) and 0.01 (−0.21, 0.24), and those for the percentage change in AMH were −0.11 (−0.34, 0.11) and −0.07(−0.27, 0.14), there was no statistical difference.

**Table 5a:** Statistical characteristics of different vaccine manufacturers in the 2-dose group. Effect of vaccine dose manufacturer on the difference in AMH and the percentage change in AMH in the two-dose group.

Variables	Sinopharm (n = 129)	Sinovac (n = 153)	Sinopharm + Sinovac (n = 244)	P-value 1
Age (years)	32.36 (28.89,36.92)	33 (29.33, 37.33)	32.42 (28.5, 37.17)	0.472
First AMH (ng/mL)	2.72 (1.1, 4.21)	2.39 (1.18, 3.45)	2.39 (1.04, 4.75)	0.771
Last AMH (ng/mL)	2.52 (0.88, 4.03)	1.95 (1.05, 3.57)	2.14 (0.92, 4.29)	0.697
Time interval between AMH examinations (days)	342 (238, 426)	333 (229, 436)	335 (239.5, 435.5)	0.935

^*^adjusted factors: Age, First AMH, Time interval between AMH examinations. Shapiro-Wilk test and Kruskal-Wallis rank sum test were used for statistical analysis.

**Table 5b:** Effect of vaccine dose manufacturer on the difference in AMH and the percentage change in AMH in the two-dose group(before and after adjustment).

Parameters	Sinopharm (n = 129)	Sinovac (n = 153)	Sinopharm + Sinovac (n = 244)	P-value 1
The difference in AMH (ng/mL)	−0.16 (−0.55, 0.39)	−0.1 (−0.61, 0.24)	−0.15 (−0.74, 0.36)	0.906
Crude B (95% CI)	–	−0.06 (−0.32, 0.2)	−0.01 (−0.24, 0.22)	
Adjust B (95% CI)	–	−0.05 (−0.3, 0.2)	0.01 (−0.21, 0.24)	
The percentage change in AMH (%)	−8 (−27, 23)	−7 (−29, 15)	−9 (−32, 18)	0.798
Crude B (95% CI)	–	−0.11 (−0.34, 0.11)	−0.08 (−0.28, 0.13)	
Adjust B (95% CI)	–	−0.11 (−0.34, 0.11)	−0.07 (−0.27, 0.14)	

The difference in AMH:last AMH – first AMH; The percentage change in AMH:(last AMH – first AMH)/first AMH. Rank sum test, regression analysis, Shapiro-Wilk test and Kruskal-Wallis rank sum test were used for statistical analysis.

DFor requirements for a specific article type please refer to the Article Types on any Frontiers journal page. Please also refer to Author Guidelines for further information on how to organize your manuscript in the required sections or their equivalents for your field^1^.

## 4 Discussion

Indicators reflecting ovarian reserve include inhibin B, estradiol (E2), FSH, etc. However, these indicators are affected by the menstrual cycle (18). AMH is produced by stratum granulosum cells of small ovarian follicles and is not affected by the dominant follicle. Therefore, the circulating level of AMH is unaffected by the menstrual cycle and can be used to measure ovarian follicular reserve. So, they are now considered the preferred measure for ovarian reserve assessment (19–22). As AMH testing is not typically included in routine gynecological examinations, it is generally conducted in most hospitals only when female patients present with symptoms indicative of abnormal ovarian function, such as insomnia, hyperhidrosis, or infertility related to ovulation. Consequently, in numerous retrospective studies, establishing a control group with normal AMH levels poses a significant challenge. However, this research relies on a sizeable Grade 3A provincial obstetrics and gynecology hospital, where the reproductive center is the main specialty. In order to screen for the causes of infertility, women visiting the reproductive center at this hospital undergo routine AMH testing, resulting in a substantial collection of samples with normal AMH levels. This includes women with other fertility issues, such as uterine adhesions and blocked fallopian tubes, who also seek treatment at the reproductive center. Consequently, this pool of patients provides the source of the research samples included in this study. Therefore, in this study, AMH was chosen as the indicator of ovarian reserve. Retrospective analysis from different angles was applied to investigate the effect of the inactivated COVID-19 vaccine on AMH levels among women. The results showed that the inactivated vaccine in China did not affect the AMH levels in women.

As a member of the TGF-β superfamily (23–25), AMH follows the classical SMAD signal transduction pathway to transmit its biological information. In the case of COVID-19 virus infection, the lungs and other affected organs trigger an inflammatory response, and in this inflammatory microenvironment, the expression of transforming growth factor β (TGF-β) is significantly increased. In theory, when the TGF-β signaling pathway is overactive, the activity or effective concentration of the Smad protein may encounter some threshold or saturation state, which prevents the Smad protein from receiving more upstream signal input or efficiently transmitting the signal further to the nucleus. If this hypothesis is true, then during COVID-19 infection, AMH may be affected by a receptor shared with the inflammatory mediator TGF-β, and interestingly, studies have shown that AMH does change significantly during COVID-19 infection (26, 27). In addition, it is worth noting that the severity of COVID-19 disease is generally thought to be related to sex (28); After COVID-19 infection, women produce fewer inflammatory factors than men (28). And mortality rates are observed to be higher in males compared to females, which suggests that premenopausal status may confer some protection against COVID-19 infection (29), This protective effect may be attributed to AMH competitively occupying a greater number of Smad receptors, and consequently, AMH may be less able to exert its effects because of this competitive binding.This may be explained from the perspective of alleviating the inflammatory response, which in turn demonstrates the association of AMH with COVID-19 infection. However, there is no conclusive evidence to confirm this saturation property of Smad protein, and more rigorous experimental studies are needed to verify this hypothesis.

Whether vaccines have the same effect on AMH levels as viruses is equally essential. This study used univariate and multivariate analyses to investigate whether the vaccine affected AMH. First, compared with the control group, the two- and three-dose groups showed no statistically significant difference in the difference and percentage change in AMH. Next, after adjusting for the first AMH, age, and time interval between AMH examinations by using a generalized linear model, no statistically significant difference in the difference and percentage change in AMH was observed among the three groups. These results suggested that different inactivated vaccine doses did not affect AMH.

A prospective study has been conducted to determine whether mRNA vaccines affect AMH. Statistical analysis of AMH levels in subjects before and after the first vaccination and three months after that study showed that AMH levels did not change significantly before and after mRNA vaccination (12). In particular, the authors mentioned that AMH changes may occur after three months or longer and require further long-term follow-up. Therefore, this study was also designed to investigate the effect of time interval after vaccination on AMH. In the two-dThe author(s) declare that fose group with the largest sample size, the patients were divided into four groups (0–60 days, 61–120 days, 121–180 days, and more than 180 days) according to the time interval from the last vaccine dose injection to the last AMH examination. The results of multivariate analysis showed no significant difference in AMH difference and percentage change of AMH in each group when the data within 0–60 days were used as a reference. This suggests that AMH levels did not change significantly after vaccination, at least during the time interval of this study. As the novel coronavirus is a recently emerged virus, it is currently unfeasible to collect samples at longer intervals to study the effect of time intervals post-vaccination on AMH levels. Our findings indicate that AMH levels remained relatively stable beyond a six-month period following vaccination. Our future research will track AMH fluctuations over a more extended duration.

The inactivated vaccines commonly administered to the Chinese population are those manufactured by Sinopharm and Kexing. The vaccination authorities do not have strict regulations on whether the manufacturer of the second dose of vaccine should be the same as the first dose, resulting in some of the population receiving vaccines from different manufacturers. Therefore, information on vaccine manufacturers was collected, and subgroup analysis was performed. The results suggested that the vaccine manufacturers did not affect the AMH level.

In conclusion, the inactivated COVID-19 vaccine, including the different vaccine doses, the time interval after vaccination, and the different vaccine manufacturers, did not affect AMH. This is consistent with the results of previous studies on the effects of other types of COVID-19 vaccines on human reproduction and female fertility. In 2022, Mohr-Sasson et al. found that ovarian reserve, as assessed by serum AMH levels, was not altered 3 months after SARS-CoV-2 mRNA vaccination (12). In 2023, Another prospective study found that although menstruation in adolescent girls may be affected by the COVID-19 mRNA vaccine, ovarian reserve did not appear to be impaired, as estimated by AMH (13).In a prospective cross-sectional study in Turkey, vaccination with COVID-19 mRNA was found to have no effect on AMH levels (30). The present study was a retrospective study to examine whether inactivated vaccines produce changes in female AMH levels in Chinese inactivated vaccine recipients. In some existing prospective analyses, due to the effect of ethics and policies actively promoting vaccination, a blank control group without vaccination was not set up (12). In the present study, a large number of samples that did not receive vaccine due to social or health factors were collected for blank control analysis, which significantly increased the credibility of the results. In addition, for the first time, this study provides a separate analysis of populations who were offered inactivated vaccines of different doses and different manufacturers. As a result, our study provides richer and more credible data on the effects of vaccines on AMH level in women.

Although AMH is widely used as a representative marker of ovarian function, studies on AMH alone to reflect the impact of COVID-19 vaccines on female reproductive capacity are far from sufficient. In fact, researchers have conducted different studies to understand the impact of various COVID-19 vaccines on female reproduction. First of all, menstruation is an essential physiological phenomenon in women of reproductive age, and the results of a study from the United States on the relationship between menstrual cycle length and COVID-19 vaccination show that the change in menstrual cycle after vaccination is less than 1 day (31). Another study examining the relationship between multiple types of COVID-19 vaccines worldwide and menstrual cycle length further found that multiple types of COVID-19 vaccination (such as mRNA vaccine, inactivated vaccine, etc.) are not associated with menstrual cycle length (32). Second, pregnancy is the most direct manifestation of average female reproductive capacity. An Internet-based pre-pregnancy cohort study in the United States found that COVID-19 vaccination had no significant correlation with the pregnancy rate of either party, and COVID-19 vaccination did not harm the fertility of either party (33). Researchers are also concerned about whether the vaccination of the COVID-19 vaccine will have an impact on assisted reproduction. A study examining women undergoing *in vitro* fertilization revealed that administration of China’s novel coronavirus inactivated vaccine did not impact key parameters in the *in vitro* fertilization process, including the number of oocytes retrieved, the implantation rate, and the sustained pregnancy rate (34). These studies, together with this and other studies on the effects of COVID-19 vaccines on AMH, provide evidence that vaccination does not have an impact on women’s reproductive health.

By analyzing a substantial amount of data, this study conclusively demonstrates that there is no basis for concern regarding reproductive health safety following administration of China’s inactivated COVID-19 vaccine. The study’s findings hold significant clinical relevance. Firstly, it dispels prevalent societal apprehensions and misconceptions about the safety of COVID-19 vaccines, thereby alleviating the need for women planning pregnancies to postpone their family planning due to vaccination concerns. Secondly, it offers a valuable perspective for women experiencing long-term infertility, suggesting that their infertility may stem from factors unrelated to vaccination.

However, this study has some limitations. Compared with those prospective studies, the age distribution of the samples in each group, the time interval between vaccinations, and the time interval between AMH examinations could not be strictly controlled. In particular, AMH is greatly affected by time factors, and AMH was measured over a long time span in this study, which may bring some errors to the results of the study. This study might benefit from additional sensitivity analyses to account for potential confounding variables or different age groups. So, a multicenter study with a larger sample size is recommended. In addition, a study by Rasa Khodavirdilou in 2022 found that AMH fluctuates significantly with the change in the menstrual cycle and that AMH at the stage of ovulation is recommended as a research indicator in clinical research on AMH (35), which may bring particular information bias to the results of this study.

## 5 Conclusion

This study demonstrated that the COVID-19 inactivated vaccine did not affect AMH levels in Chinese women from the number of doses, the manufacturer and the time interval after vaccination. The findings of this study present compelling clinical proof in support of the safety of COVID-19 vaccination, with particular emphasis on the reproductive health safety of Chinese women. These findings effectively address the concerns that vaccines might adversely affect AMH levels. Consequently, healthcare professionals can confidently recommend the COVID-19 vaccine to female patients without hesitation regarding its potential negative impact on fertility. This not only boosts public trust in vaccination and increases vaccination rates but also serves as a crucial measure to safeguard public health and promote women’s health and well-being.

## Data Availability

The original contributions presented in the study are included in the article/supplementary material. Further inquiries can be directed to the corresponding authors.

## Footnotes

1 For Original Research articles, please note that the Material and Methods section can be placed in any of the following ways: before Results, before Discussion or after Discussion.
